# Flowering also has to end: knowns and unknowns of reproductive arrest in monocarpic plants

**DOI:** 10.1093/jxb/erad213

**Published:** 2023-06-02

**Authors:** Vicente Balanzà, Paz Merelo, Cristina Ferrándiz

**Affiliations:** Instituto de Biología Molecular y Celular de Plantas, Consejo Superior de Investigaciones Científicas - Universidad Politécnica de Valencia, 46022, Valencia, Spain; Instituto de Biología Molecular y Celular de Plantas, Consejo Superior de Investigaciones Científicas - Universidad Politécnica de Valencia, 46022, Valencia, Spain; Instituto de Biología Molecular y Celular de Plantas, Consejo Superior de Investigaciones Científicas - Universidad Politécnica de Valencia, 46022, Valencia, Spain; University College Dublin, Ireland

**Keywords:** APETALA2, auxin, cytokinin, death hormone, end of flowering, FRUITFULL, inflorescence meristem, meristem arrest, proliferative arrest

## Abstract

All flowering plants adjust their reproductive period for successful reproduction. Flower initiation is controlled by a myriad of intensively studied factors, so it can occur in the most favorable conditions. However, the end of flowering is also a controlled process, required to optimize the size of the offspring and to maximize resource allocation. Reproductive arrest was described and mainly studied in the last century by physiological approaches, but it is much less understood at the genetic or molecular level. In this review, we present an overview of recent progress in this topic, fueled by highly complementary studies that are beginning to provide an integrated view of how the end of flowering is regulated. In this emerging picture, we also highlight key missing aspects that will guide future research and may provide new biotechnological avenues to improve crop yield in annual plants.

## Teaser

An integrated view of recent progress in the study of the control of the end of flowering in monocarpic plants is presented.

## Introduction

All living beings must optimize their reproductive success. On this premise, organisms have adopted different reproductive strategies to ensure the viability of the species by the production of an optimal number of descendants. In general terms, the two most prevalent strategies in nature are iteroparity (when the individual reproduces in several cycles) and semelparity (when it reproduces just in one) (Stearns, 1976; Charlesworth, 1994). In plants, those categories are represented by the terms polycarpy and monocarpy. When growth conditions are favorable, allowing access to resources, optimal temperature conditions, pollination vectors, etc., plants start reproductive development, which in angiosperms involves initiating the production of flowers from an inflorescence meristem (IM). However, the flowering period also must be terminated to optimize the size and viability of the offspring. Thus, after the production of a certain number of flowers, the IMs arrest, ending the production of fruits and seeds. In monocarpic plants, IM arrest triggers (or at least usually precedes) a global senescence program that ends the life of the plant, while, in polycarpic plants, the senescence program does not affect the entire plant, which can resume reproductive development from other meristems when growth conditions are favorable again in following seasons (Molisch, 1938; Whaley, 1939; Leopold *et al.*, 1959).

The regulation of both flowering initiation and termination, in addition to huge importance for fitness and reproductive success, also has a major impact in agronomical systems. Floral transition has been intensely studied by plant researchers, and a vast amount of knowledge has been gathered in the past few decades on the genetic, environmental, and molecular factors across model and crop species. In contrast, and somehow shockingly, the regulation of flowering termination has been a highly overlooked and neglected topic, which has gained some momentum only in the last few years. Now, a wealth of data is finally becoming available, although still almost exclusively in Arabidopsis. Thanks to a number of inspiring studies that tackle the subject from different experimental approaches, we are learning about the physiology, the genetic regulation, the hormonal pathways involved in the process, and other signaling mechanisms that control the termination of the reproductive phase in this model species.

Here, we review the major recent advances in the topic and propose new directions and perspectives for future research.

## The early studies: a physiological view and the death hormone

Several works dating as early as the first decades of the 20th century described that many species of monocarpic plants (including legumes, tomato, grasses, etc.), after a period of continuous flowering, undergo the arrest of the proliferative capacity of the IMs, thus ending the production of flowers, followed by a global senescence program. These studies showed how fruit and seed development are a major factor promoting this arrest, and several hypotheses were proposed to explain how it is established, such as source–sink relations between developing fruits and the meristems, or the existence of a seed-derived signal that, at least in some species, was even demonstrated to be graft transmissible, and thus came to be called the ‘death hormone’ (Proebsting *et al.*, 1977; Kelly and Davies, 1988; Noodén and Leopold, 1988; Wilson, 1997). Interestingly, proliferative arrest controlled by the putative fruit/seed-derived signal occurred in plants both with determinate [where the IM eventually adopts floral identity and flowering may proceed in other axillary meristems (AMs)] and with indeterminate inflorescences (where the meristem remains indeterminate but stops its activity to end the production of new flowers), indicating that the termination of flower production was somehow coordinated at the whole-plant level and not dependent on the fate (determinate/indeterminate) of the shoot apical meristems (SAMs) (Murneek, 1926, 1932; Lockhart and Gottschall, 1961; Lindoo and Noodén, 1977; Biswas and Choudhuri, 1980; Reid, 1980; Mauk *et al.*, 1990; Wilson, 1997; Noodén and Penney, 2001).

A systematic study carried out in the 1990s on the model plant *Arabidopsis thaliana*, also monocarpic, identified some factors that affect the moment at which proliferative arrest occurs (Hensel *et al.*, 1994). This work corroborated that production of seeds was a major force to stop flowering, since Arabidopsis mutants with highly reduced fertility extend IM activity for longer than fertile plants. In addition, once proliferative arrest has happened, the IM could be reactivated by fruit removal, supporting the repressive long-distance effect of seeds on meristem activity. This study also showed that the end of flowering was controlled genetically, and explored the possible contribution of different hormonal pathways, but did not succeed in identifying the nature of the ‘death hormone’ (Hensel *et al.*, 1994).

A more recent study has revisited the work of Hensel *et al.*, adding detailed experiments that greatly help to understand the process at the physiological level (Ware *et al.*, 2020). Among major findings, it shows how the seed effect appears to be active only when inflorescences have acquired the ‘commitment’ to arrest, suggesting that the mechanism that mediates this effect could be dependent on the age of the inflorescence. A second important finding was that auxin exported from fertile fruits is an important component of the signaling mechanisms triggering the arrest (Ware *et al.*, 2020). Complementing this work, a recent study describes that the inflorescence arrest can be separated into two phases (Walker *et al.*, 2023): one affecting the cessation of the formation of new floral primordia (meristem arrest) which occurs first and would be highly dependent on cytokinin (CK) activity; and a second one that affects the progression of development of already formed floral primordia. In this work, it is described that after meristem arrest, the already formed floral primordia continue their development, giving rise to mature flowers, but, at a certain moment, the formed floral primordia at developmental stage 9 and below suffer the second and last step of inflorescence proliferative arrest (floral arrest) where CK and auxins would play a major role. Thus, according to this work, auxins would only be involved in this second phase (Walker *et al.*, 2023).

## The genetic control of inflorescence meristem arrest

### Maintenance of the indeterminate inflorescence meristem

In Arabidopsis, the SAM contains the niche of the stem cells that sustain all the growth of the aerial part of the plant. After floral transition, the vegetative SAM becomes the IM, initiating the production of floral primordia instead of leaves in its flanks, as well as sustaining the elongation of the stem in its base. Then, the IM is the structure that sustains the production of all the flowers of the inflorescence. The continuous formation of flowers by the IM requires that it remains in an undifferentiated state, providing new cells that allow the formation of new floral primordia. The major genetic functions that preserve meristem structure and functionality have been extensively characterized. In brief, the WUSCHEL (WUS)–CLAVATA (CLV) feedback loop maintains and controls the number of stem cells in the meristem. Meristem maintenance is regulated by the negative feedback loop between WUSCHEL (WUS) and the CLV module. WUS promotes the expression of *CLV3* in the stem cell population, while CLV3 (a secreted peptide) binds the CLV1/CLV2/CORYNE receptors that mediate the repression of *WUS* (Brand *et al.*, 2000; Schoof *et al.*, 2000; Müller *et al.,* 2008). This mutual regulation ensures that the population of stem cells in the meristem is maintained in the right proportion. Together with the *WUS–CLV* genes, *SHOOT MERISTEMLESS* (*STM*) participates in the homeostasis of the meristem avoiding excessive cell differentiation (Long *et al.*, 1996). In addition, the phosphatidylethanolamine-binding protein (PEBP) TERMINAL FLOWER1 (TFL1) confers indeterminate IM identity. In a *tfl1* mutant, the IM is transformed into a floral meristem very early in development, due to the ectopic expression of the floral identity genes *LEAFY* and *APETALA1* (Bradley *et al.*, 1997).

**Fig. 1.**
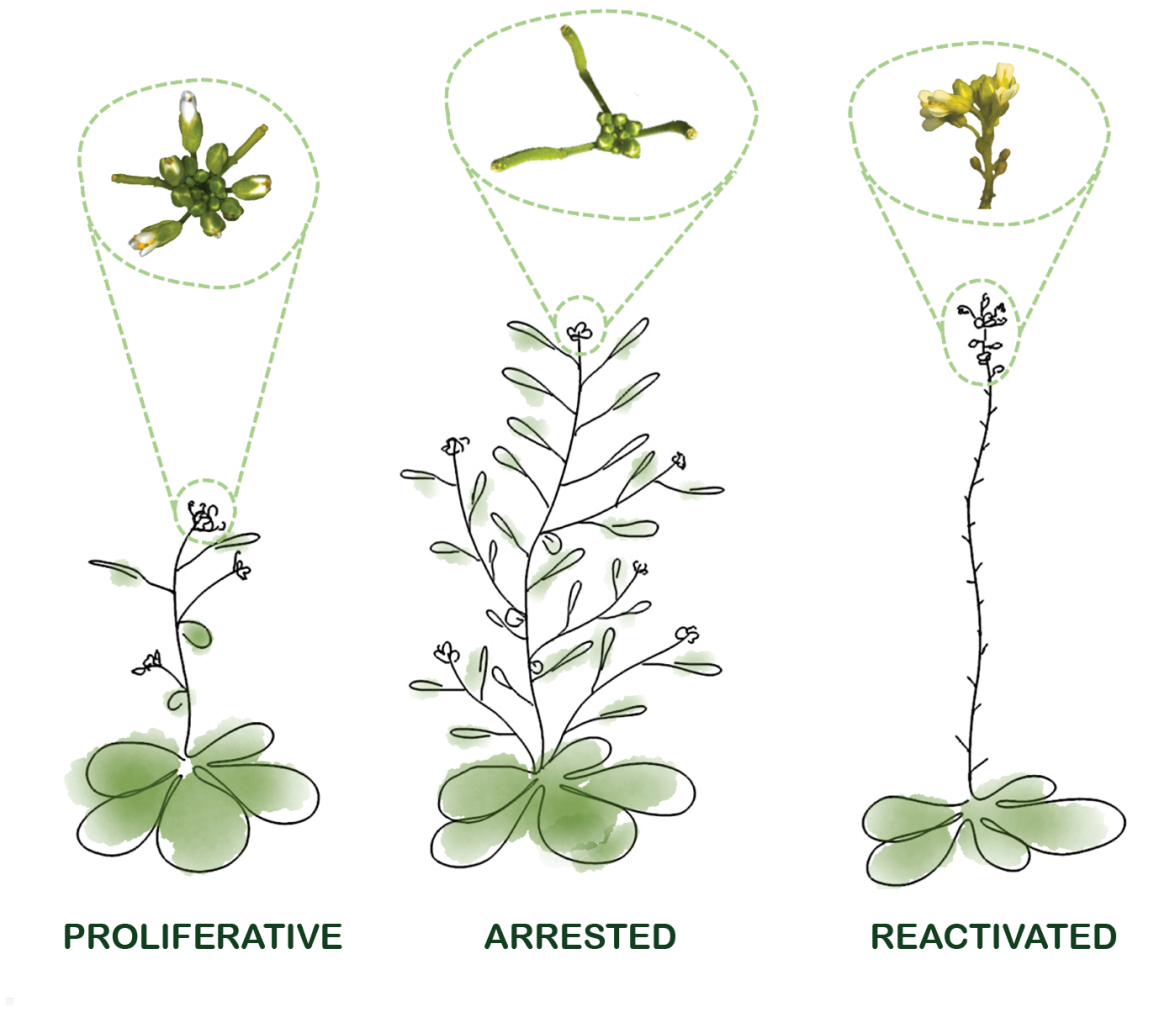
Morphology of the inflorescence apex in Arabidopsis plants actively proliferating (flowering), arrested (at the end of flowering), or reactivated by pruning of developed fruits after arrest. Cartoons below show the overall aspect of the plants, while in the circled areas, pictures of inflorescences apexes are shown. Note the cluster of unopened buds in arrested inflorescences, in contrast to the developing fruits in basal positions of the stem. In reactivated inflorescences, at least some of these buds do not develop further, and new flowers arise from the reactivated meristem.

During the flowering period, the IM becomes smaller with time, due to a decrease in cell size and number, but maintains its structure and function (Y. Wang *et al.*, 2020; Merelo *et al.*, 2022; Walker *et al.*, 2023). The IM arrest observed at the end of flowering, understood as the moment when no more flowers are produced by the IM, shows many similarities to dormant AMs at the transcriptomic level (Wuest *et al.*, 2016; Martínez-Fernández *et al.*, 2020). At this time point, no more floral primordia are generated and those already formed arrest their development (stage 9 and below) (Walker *et al.*, 2023). Inflorescences that have experienced proliferative arrest exhibit a distinctive morphology, with a sharp contrast between the stem supporting developing fruits and the cluster of unpollinated buds that do not develop further (Fig. 1). The inflorescence arrest is eventually followed by the activation of a senescent program that in Arabidopsis and other monocarpic plants is extended to the whole plant (Y. Wang *et al.*, 2020).

In the arrested IM, WUS activity is no longer detected in the organizing center (Balanzà *et al.*, 2018; Goetz *et al.*, 2021; Merelo *et al.*, 2022). *wus* mutants are characterized by meristem exhaustion, due to non-renewal of stem cells, but, interestingly, in arrested IMs, the absence of WUS does not imply the extinction of the meristem. Actually, the arrested IM expresses the *CLV3* and *STM* genes until the initiation of the senescence program, indicating that stem cells are still present up to this phase (Balanzà *et al.*, 2018; Y. Wang *et al.*, 2020; Goetz *et al.*, 2021). It has been described that in addition to WUS, STM can positively regulate the expression of *CLV3*, albeit in a way mostly dependent on WUS (Brand *et al.*, 2000). As no other WUS-independent activators of *CLV3* are known, additional factors yet to be identified could act after proliferative arrest to maintain *CLV3* expression. It is also noteworthy that cell division events are strongly reduced prior to *WUS* repression (Merelo *et al.*, 2022). When arrested meristems are reactivated by pruning of the fruits, *WUS* expression immediately restarts in the IM to sustain the development of the new floral primordia and is associated with active cell division (Merelo *et al.*, 2022). It could be interesting to check if the reactivation ability of the meristem depends on the presence of STM and CLV3, and also if the unusually uncoupled WUS/CLV3 regulation could be linked to the proposed separate events of IM arrest and blockage of flower formation (Walker *et al.*, 2023) or even to the action of the putative seed-derived signal in some way. In any case, further investigations are needed to explore these hypotheses.

### The FUL–AP2 pathway

**Fig. 2.**
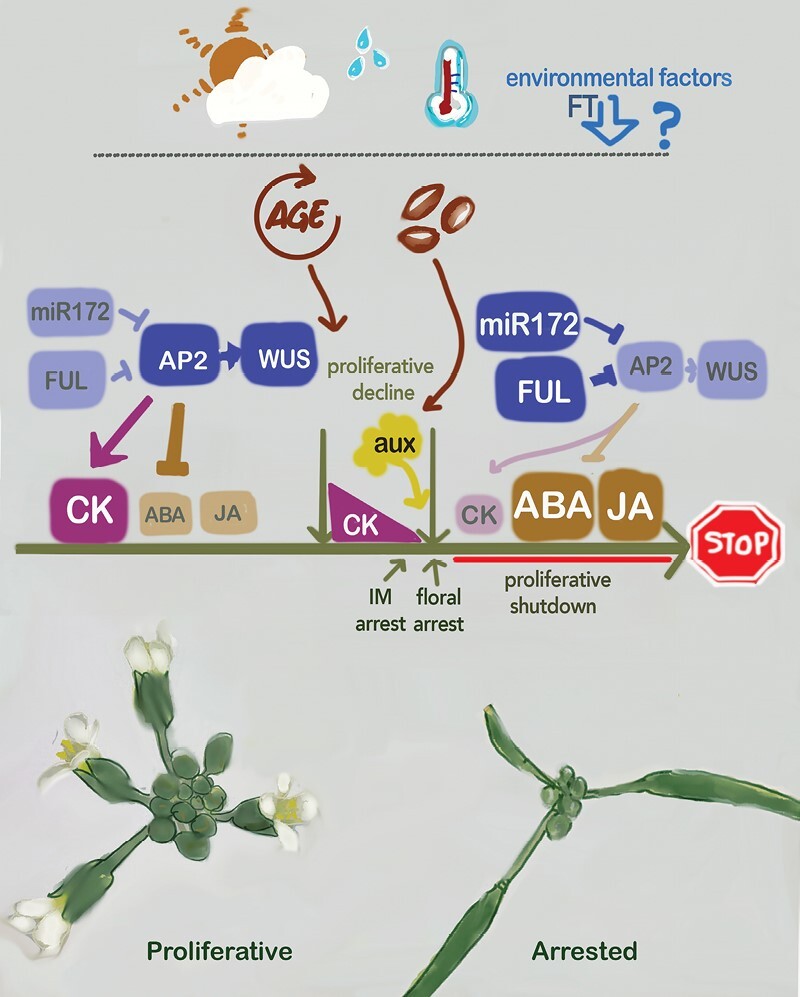
Summary of factors influencing proliferative arrest. Environmental factors affect the timing of the beginning and the end of flowering, partly through FT, although their precise contribution to proliferative arrest is still largely uncharacterized. Endogenous clues such as age or, more importantly, the production of seeds are major players in the control of the process. In inflorescences in the proliferative state, at earlier stages of the reproductive phase, CK signaling and transcription factors maintaining the activity of the meristem (AP2, WUS) are active, while age-related factors such as miR172 or FUL, negative regulators of AP2, are present at relatively low levels. At the end of the flowering phase, corresponding to the proliferative decline described by Merelo *et al.* (2022), CK signaling, AP2 and WUS levels decrease, in part by the negative regulation exerted by FUL and miR172, which accumulate with age. WUS decline is accompanied by the cessation of meristem activity, and in the shutdown phase (inflorescence arrest) WUS is no longer detected. In addition, the decreasing levels of AP2 cause high ABA and JA signaling, inducing a dormant-like state of the meristem. Auxin exported from fruits has a strong influence on meristem activity, and it has been proposed that they act to promote the floral arrest phase (Walker *et al.*, 2023).

The first evidence that the proliferative arrest was controlled at the genetic level was shown by the analysis of *fruitfull* (*ful*) mutants. Balanzà *et al.* (2018) described that *ful* plants did not arrest like wild-type plants, independently of the number of seeds produced. The increased reproductive period of *ful* mutants is dependent on the presence of the *APETALA2* (*AP2*) and *AP2-like* genes. Additionally, it was also demonstrated that miR172, a repressor of AP2-like activity, plays an important role in the control of the proliferative arrest. The *ap2-170* mutant, where the miR172-binding site on the AP2 transcript is mutated, presents a delayed end of flowering. The model proposed for the control of the proliferative arrest hypothesized that the high levels of *FUL* and *miR172*, controlled in an age-dependent way (Wang *et al.*, 2009; Wu *et al.*, 2009), directly repress the expression of *AP2-like* genes [*AP2*, *SCHNARCHZAPFE*N (*SNZ*), *TARGET OF EARLY ACTIVATION TAGGED 1* (*TOE1*), and *TOE3*], promoters of meristem activity through the activation of *WUS* (Balanzà *et al.*, 2018) (Fig. 2). The fine regulation of *AP2* is a crucial step in all kinds of reproductive meristems. *AP2-like* genes are regulated at different levels: transcriptional, post-transcriptional, and translational (Aukerman and Sakai, 2003; Chen, 2004; Schwab *et al.*, 2005; Würschum *et al.*, 2006; Lian *et al.*, 2021; ÓMaoiléidigh *et al.*, 2021; Sang *et al.*, 2022), and they have been shown to work redundantly in different processes (Yant *et al.*, 2010; Jung *et al.*, 2014). In addition, AP2 is able to repress itself as well as its regulators *miR172* and *FUL* (Yant *et al.*, 2010; ÓMaoiléidigh *et al.*, 2021). Lastly, based on the observation that FUL is able to interact with several chromatin-remodeling factors (Smaczniak *et al.*, 2012; van Mourik *et al.*, 2023), it has been proposed that the combined action of FUL and miR172 could mediate the epigenetic silencing of *AP2-like* (ÓMaoiléidigh *et al.*, 2021).

FUL is required to induce the proliferative arrest as IMs of *ful* mutants never arrest. This phenotype is even better observed in the absence of seeds, where the wild-type IM does not undergo proliferative arrest but eventually differentiates into a terminal flower (Hensel *et al.*, 1994; Balanzà *et al.*, 2018; Merelo *et al.*, 2022), while *ful* mutants continue producing flowers at a low rate indefinitely. Moreover, the overexpression of *FUL* stops flower production, transforming the IM into a terminal flower (Ferrandiz *et al.*, 2000; Balanzà *et al.*, 2014). The absence of miR172 produces enlarged IMs that are active for longer (Lian *et al.*, 2021; ÓMaoiléidigh *et al.*, 2021; Sang *et al.*, 2022), while the overexpression of miR172 produces plants with an early meristem differentiation and low flower production (Balanzà *et al.*, 2018, 2019). On the other hand, the induction of an miR172-resistant version of *AP2* is able to reactivate an arrested SAM and avoid or delay the proliferative arrest (Balanzà *et al.*, 2018; Martínez-Fernández *et al.*, 2020), while the *ap2* mutant develops smaller IMs and shows a shorter flowering period (Balanzà *et al.*, 2018; Sang *et al.*, 2022). The balance between FUL/miR172 and AP2 seems to be a key element in the temporal maintenance of the IM and timing of proliferative arrest. However, even though both FUL and miR172 control AP2 levels and their mutants extend the activity of the IM, they appear to act on different aspects of meristem dynamics. *ful* mutants are affected mainly in the final arrest of the meristem, controlling the timing of this event without affecting meristem size (Merelo *et al.*, 2022), while *miR172* mutants clearly have an effect on IM size, possibly affecting the cell number/division in the meristem (Lian *et al.*, 2021; Sang *et al.*, 2022). In agreement with these observations, the double mutant *ful ap2-170* (*AP2* allele resistant to miR172) shows a phenotype that could be considered as an additive effect of both mutations, lacking proliferative arrest plus an increase in meristem size that also has an impact of the number of flowers produced (Balanzà *et al.*, 2018).

### Downstream of the death hormone and AP2: the routes at work that characterize meristem arrest

Two studies characterizing the transcriptomic landscape in IMs at different stages shed light on the major routes involved in proliferative arrest. Wuest *et al.* (2016) compared actively proliferating IMs with arrested meristems and with meristems that had been reactivated by removing all fruits in the inflorescence. The arrested meristems have transcriptomic signatures associated with low mitotic activity, responses to growth- and branching-inhibitory hormones, and the induction of stress and senescence programs, but, interestingly, also a reduction of reactive oxygen species (ROS), a feature of dormant buds at least in some perennial species (Signorelli *et al.*, 2018; Considine and Foyer, 2021). After reactivation by removal of the seed-derived signal, the meristems recover a transcriptomic profile similar to that of the active IMs before arrest (Wuest *et al.*, 2016)

Martinez-Fernández *et al*. (2020) showed that arrested meristems could also be reactivated by inducing *AP2* expression in the apex and studied the transcriptomic response to this induction in the IM. AP2 induces opposite changes in gene expression to those observed in arrested meristems, and similar to those described in the study by Wuest *et al.* (2016) caused when meristems are reactivated by fruit removal: abscisic acid (ABA) responses are repressed, while CK responsiveness is induced; stress- and senescence-related genes are also repressed; and several genes involved in the response to temperature or to light quality are repressed by AP2 and accumulate in arrested meristems, suggesting that environmental conditions could also be influencing proliferative arrest timing.

Altogether, these studies indicate that arrested meristems experience a process related to bud dormancy, somehow providing a basis to account for their capacity to reactivate when arrest-promoting signals are eliminated. A second attractive idea coming from these works is that, given that highly similar responses in the meristem are caused either by removing the seed signal or by *AP2* induction, *AP2-like* genes could be in some way integrating the information coming from seeds and not only the age-related cues. Moreover, ~25% of the genes regulated by AP2 in the meristem have been identified previously as putative direct targets of this factor by ChIP-seq (Yant *et al.*, 2010), suggesting that AP2 could be an orchestrator of the different routes required for meristem arrest at the end of the flowering phase.

## A hormone cocktail to trigger and modulate arrest

The characterization of proliferative arrest carried out by different studies in the last few years has uncovered how different hormone signaling pathways are likely to be players in the integration of endogenous cues, physiological conditions, and environmental signals to trigger arrest and to coordinate the required responses in the meristem. Certain hormones have been suggested to be part of the seed-derived signal (González-Suárez *et al.*, 2020; Ware *et al.*, 2020; Goetz *et al.*, 2021), to be important for the acquisition of the competence to perceive this and other signals by the plant (Martínez-Fernández *et al.*, 2020; Merelo *et al.*, 2022), or to consolidate the dormant-like state of the arrested meristems (Wuest *et al.*, 2016; Martínez-Fernández *et al.*, 2020). In the following sections, we will review the evidence recently gathered to support these proposed mechanisms and other emerging hypotheses on additional signaling pathways.

### Auxin transport from fruits promotes inflorescence arrest

As previously mentioned, early works pointed to a systemic signal (the death hormone) from the fruits or seeds as a potential factor triggering proliferative arrest (Noodén and Leopold, 1988; Hensel *et al.*, 1994; Wilson, 1997). It has been extensively reported that fruits and seeds produce high levels of auxin in different species (Gustafson, 1939; Matilla, 2020; Godoy *et al.*, 2021; Zhang *et al.*, 2021). Moreover, the process of activation of dormant AMs is partially regulated by the export of auxin from the AM to the stem (Tamas *et al.*, 1981), and arrested IMs resemble, at the transcriptional level, dormant AMs (Wuest *et al.*, 2016; Martínez-Fernández *et al.*, 2020). Considering these studies, Ware *et al.* (2020) proposed that auxin exported from fruits could be the proliferative arrest-promoting signal. In this work, it was shown that auxin content in the exudates from fertile fruits were much higher than in those from sterile fruits and also that application of auxin to fruits of a sterile mutant or to pedicels of removed fruits could induce arrest. Moreover, mutants with impaired auxin transport in the stem showed a delayed arrest. These experiments led the authors to propose a model where auxin export from developing fruits induces proliferative arrest by disrupting polar auxin transport in the apical region of the stem. This work also showed a positional effect of fruits in the promotion of arrest, more effective in proximal positions to the IM, and that the inflorescence has to acquire ‘competence’ to respond to this signal in later phases of the inflorescence development (Ware *et al.*, 2020) (Fig. 2). Later on, the distinction of the two phases of inflorescence arrest (IM and floral) proposed in Walker *et al.* (2023), where the timing of these two events is calculated based on morphological markers, appears to be in conflict with the possible role of auxin exported from fruits in the arrest of the IM. These observations encourage a more detailed investigation of the precise characterization of auxin dynamics in inflorescence arrest to better understand the mechanisms that trigger the end of the reproductive phase and the nature and mode of communication with the fruits.

In the same direction, a different study (Goetz *et al.*, 2021) showed by quantification of radiolabeled indole-3-acetic acid (IAA) in stem segments that auxin transport decreases gradually at the apical region of the stem prior to the bud cluster observation. Here, Goetz *et al.* (2021) proposed that such a decline of auxin transport would interrupt auxin canalization in the apical region of the stem, and extend the previous model by showing that arrest correlates with an increase in auxin response in the apical region (Fig. 2). Moreover, the authors theorize that these changes in auxin response would repress auxin transport in the apical region. However, an increase in the MONOPTEROS (MP) expression in this region is observed, that is somehow in conflict with the positive feedback between auxin response (MP- and DR5-mediated) and transport that has been previously reported in the meristem (Heisler *et al.*, 2005; Bhatia *et al.*, 2016). Again, more data to delve into the molecular mechanism under the hypothesis proposed by Goetz *et al.* (2021) will be required.

These works may suggest that auxin is the long-sought death hormone. However, there are still open questions that need to be addressed before this can be proven. It is possible that the effect of the fruit/seed-derived auxin may be indirect and that a second molecule could be produced. If indirect, what and where is this messenger induced by auxin? Ware *et al.* (2020) suggest that a second messenger is not produced in the fruits and that it could be produced in the stem, but still more work is needed to define the site of auxin action. Finally, it is unclear how the proposed competence of the meristem to respond to the signals from seeds can be correlated with changes in auxin transport in the stem. To determine the molecular basis underlying a potential effect of auxin in the acquisition of such competence to arrest or whether auxin acts downstream of arrest competence factors will also help to understand the precise mode of action of auxin in the process.

### Repression of cytokinin-related pathways triggers proliferative arrest

Studies on CK control of plant development showed alterations in arrest time in CK-related mutants. Gain-of-function mutants of genes encoding the cytokinin receptors AHK2 and AHK3 (ARABIDOPSIS HISTIDINE KINASE) flower for extended periods (Bartrina *et al.*, 2017), suggesting that negative regulation of CK activity may be required for proliferative arrest. However, these mutants display pleiotropic effects such as delayed leaf senescence, which may prolong meristem activity (Hensel *et al.*, 1994; Noodén and Penney, 2001; Wuest *et al.*, 2016), and thus do not clarify whether CK may work locally within the meristem to prevent arrest or cause systemic changes with whole-plant physiological effects.

In a subsequent work, the repression of CK-related pathways locally in the IM has been shown to be required for proliferative arrest (Merelo *et al.*, 2022). In particular, this study shows, by monitoring with high spatiotemporal resolution meristem activity and cell proliferation markers, that proliferative arrest involves a coordinated temporal repression of CK signaling and CK-dependent processes, such as CYCLINB1;2-promoted cell division, WUS-mediated SAM activity, and SAM growth (Fig. 2). CK treatment of active and arrested apices reveals that this hormone is sufficient to prevent and revert the process, respectively. The decline of CK-related processes observed prior to proliferative arrest could be related to the competence of the meristem to perceive the seed signals proposed by Ware *et al.* (2020), but further work is needed to explore this hypothesis.

In addition, an interesting connection with the FUL–AP2 pathway has been uncovered, since CK-related signaling fails to be repressed in *ful* mutants, suggesting that FUL may promote meristem arrest via repression of these CK-related pathways. FUL’s role at the end of the reproductive period appears to be biphasic: first, FUL, together with additional unknown factors, would gradually repress CK-related processes in the IM (phase I, decline) and, later, when the cluster of arrested buds is visible, it would completely block these pathways, leading to meristem activity arrest (phase II, shutdown) (Merelo *et al.*, 2022). As discussed earlier, AP2, the other major component of the age-dependent network controlling arrest, may directly repress the negative regulators of CK signaling *KISS ME DEADLY2* and *KMD4* (Kim *et al.*, 2013). *KMD2* and *KMD4* expression increase at the moment of meristem arrest, and loss-of-function mutant and overexpression lines exhibit a delayed or early arrest, respectively. Thus, AP2 may promote *WUS* expression and meristem activity via *KMD* repression. At the end of the flowering period, the decline of AP2 activity would cause an increase in KMD protein levels, shutting down CK signaling and *WUS* expression and leading to meristem arrest. Since FUL is a repressor of *AP2*, it may regulate CK signaling through *AP2* and/or by directly regulating CK-related events (Merelo *et al.*, 2022), but more work is needed to clarify these non-excluding scenarios.

Finally, a recent study (Walker *et al.*, 2023) has extended the knowledge regarding the role of CK in proliferative arrest. In particular, the authors showed that gain-of-function mutations in *AHK2* and *AHK3* genes (*rock2* and r*ock3*, respectively) caused a differential regulation of IM and floral arrest. *rock2* displayed alterations in both IM and floral arrest, whereas *rock3* mainly affected floral arrest, which indicates that CK participates in both arrest processes. In addition, based on inflorescence or fruit removal assays, an in-depth characterization of loss-of-function *ahk2 ahk3* mutants, and quantification of CK content in fertile and sterile fruits, this work proposed that CK re-distribution between sinks (inflorescences and fruits) balances the duration of IM or flower opening activity and reproductive success. It would also be interesting to test whether seed signals could be acting directly on CK-related pathways or being integrated through the FUL–AP2 genetic pathway.

### Transcripts of abscisic acid-responsive genes accumulate in arrested meristems

Wuest *et al.* (2016) identified a set of genes related to ABA response strongly up-regulated in arrested meristems when compared with growing or reactivated-by-pruning meristems. Since many of these genes are similarly regulated in dormant axillary buds (González-Grandío *et al.*, 2013; Yao and Finlayson, 2015) and marker genes for dormant buds were among the most highly up-regulated genes in arrested meristems, it was suggested that proliferative arrest may constitute a form of meristem dormancy (Wuest *et al.*, 2016).

The same set of ABA-related genes that were up-regulated in arrested meristems (Wuest *et al.*, 2016) are down-regulated upon AP2-mediated reactivation (Martínez-Fernández *et al.*, 2020). Among these putative AP2 targets, there are genes related to ABA biosynthesis, perception, signaling, and response, including HB21 and HB53 that promote ABA accumulation and response during axillary bud dormancy (González-Grandío *et al.*, 2017). Again, the decline of AP2 activity at the end of the flowering period would lead to high levels of these HB factors and, thus, to high levels of ABA and ABA response (Fig. 2). The capacity of AP2 to reactivate arrested meristems by repressing these pathways again points to similarities between proliferative arrest and axillary meristem dormancy. Experiments beyond ABA treatments and transcriptomic analyses in the proliferative arrest context will help to determine whether the gene regulatory networks controlling both processes converge.

### Jasmonic acid and proliferative arrest

Two genetic studies have proposed that JA may indirectly or directly play a role in the control of proliferative arrest. Mutations in genes involved in JA biosynthesis, *13-LIPOXYGENASE3* (*LOX3*) and *LOX4*, caused alterations in male fertility and proliferative arrest (Caldelari *et al.*, 2011), but these effects can be considered indirect and caused by the absence of seed production. A different study showed that a mutant in the jasmonic acid (JA) co-receptor CORONATINE INSENSITIVE 1 (*coi1-37*), also male-sterile, caused dramatically extended IM activity and, unlike other sterile mutants, did not form a terminal flower (Kim *et al.*, 2013). However, this phenotype was only observed in one of the genetic backgrounds (ecotype) tested, and then it is not clear what were the relative contribution of JA and other unknown factors to the alterations in meristem arrest. In any case, this evidence suggests a potential direct effect of JA on flowering termination, maybe related to its role in promoting bud dormancy like ABA (González-Grandío *et al.*, 2017; Dong *et al.*, 2019). Additional assays aimed at characterizing the distribution of JA in the IM or the potential interaction between JA and other hormones in the meristem context will help to better understand its contribution to the control of proliferative arrest (Fig. 2).

### Other signaling molecules and proliferative arrest: sugars and reactive oxygen species

A recent study (Goetz *et al.*, 2021) proposes that the end of flowering comprises a first stage of apex growth cessation, where the apex and the first internodes below transition to a quiescent stage (quiescent 1 stage, Q1), and a second quiescent stage (Q2), where growth of the apex and the last fruits arrests. Probably, these two stages correlate in time with the two phases described by Merelo *et al.* (2022) (decline and shutdown, respectively), based on the inflorescence morphology. This work suggested that changes in sugar signaling and metabolism start at the Q1 stage in the apex and may participate in arrest. The onset of inflorescence arrest (Q1 stage) would imply the reduction of sugar signaling through glucose and the T6P (TREHALOSE 6-PHOSPHATE) pathway in the apex. Moreover, sucrose metabolism would be supressed in Q1 apices, but would increase in developing fruits at this stage. Based on these and previous pieces of evidence showing that sugars act as growth and signaling molecules (Eveland and Jackson, 2011), the authors proposed two hypotheses. First, proliferative arrest would imply a cessation of inflorescence growth that would trigger an increase in the growth potential of developing fruits (sink potential), which correlates with the previous idea of source–sink relationships in the plant (Wuest *et al.*, 2016). Second, the increase in the growth potential of developing fruits may be mediated by the arrest competence factors. It would be interesting to unveil the nature of such factors as well as the potential connection between these sugar-related changes (Goetz *et al.*, 2021), hormone-related changes (Ware *et al.*, 2020; Goetz *et al.*, 2021; Merelo *et al.*, 2022), and the acquisition of competence to arrest.

Among the ROS, H_2_O_2_ has been shown to be a fundamental signaling molecule in different developmental processes (Niu and Liao, 2016). The balance between the two ROS, O_2_·^–^ and H_2_O_2_, is required for stem cell maintenance and differentiation in the SAM (Zeng *et al.*, 2017; J. Wang et al., 2020). In particular, O_2_·^–^ promotes *WUS* expression and the maintenance of the stem cell niche, and H_2_O_2_ represses O_2_·^–^ biosythesis in the peripheral zone of the SAM, allowing differentiation. Previous studies also suggest that H_2_O_2_ may repress *WUS* expression. Both the study on proliferative arrest carried out by Wuest *et al.* (2016) and the study by Wang *et al.* (2022) showed that O_2_·^–^ was detectable in active IMs but undetectable in arrested IMs, correlating with the WUS spatiotemporal expression pattern (Balanzà *et al.*, 2018; Merelo *et al.*, 2022; Wang *et al.*, 2022). Moreover, Y. Wang *et al.* (2020, 2022) showed that H_2_O_2_ accumulated in arrested SAMs, suggesting that ROS may play a role in the control of meristem arrest (Y. Wang *et al.,* 2020). These data were also supported by a transcriptomic analysis that identify a set of ROS-related genes over-represented in arrested apices in comparison with active apices (Wang *et al.*, 2022). A future challenge will be to determine whether these additional signals (sugars and ROS) act upstream or downstream of the age-dependent FUL–AP2 module or constitute parallel pathways in the regulation of this process.

## What next? Open questions and perspectives

The study of the mechanisms controlling the end of flowering is gaining momentum. Environmental control of the process has just been addressed in an exciting study that shows how temperature and light intensity are relevant factors that impact in the duration of the reproductive phase, in a way mediated by the flowering integrator FLOWERING LOCUS T (FT) (González-Suárez *et al.*, 2023). Still, many open questions remain, and here we can propose some major gaps that need to be filled in multiple directions. What is the exact nature of the seed-derived signal and how is it perceived in the meristem? How does the crosstalk between hormonal signals work in the meristem? How is this translated into a transcriptional response? Does it involve epigenetic mechanisms? How conserved are the mechanisms and signals uncovered in Arabidopsis in other monocarpic species? And in perennials? Can we use the knowledge for biotechnological applications?

Clearly, almost endless questions can be posed at this moment, making it a lively topic where new ideas and hypotheses can be explored and tested, and we can expect to significantly increase our knowledge on the process in the next few years.
